# Managing patient safety and staff safety in nursing homes: exploring how leaders of nursing homes negotiate their dual responsibilities—a case study

**DOI:** 10.3389/frhs.2024.1275743

**Published:** 2024-01-29

**Authors:** Malin Rosell Magerøy, Carl Macrae, Geir Sverre Braut, Siri Wiig

**Affiliations:** ^1^ SHARE – Centre for Resilience in Healthcare, Faculty of Health Science, University of Stavanger, Stavanger, Norway; ^2^ Centre for Health, Innovation, Leadership and Learning, Nottingham University Business School, Nottingham, United Kingdom; ^3^ Department of Research, Stavanger University Hospital, Stavanger, Norway; ^4^ Department of Social Science, Western Norway University of Applied Sciences, Sogndal, Norway

**Keywords:** leadership, patient safety, staff safety, quality, human factors

## Abstract

**Objective:**

Within healthcare, the role of leader is becoming more complex, and healthcare leaders carry an increasing responsibility for the performance of employees, the experience and safety of patients and the quality of care provision. This study aimed to explore how leaders of nursing homes manage the dual responsibility of both Health, Safety and Environment (HSE) and Quality and Patient Safety (QPS), focusing particularly on the approaches leaders take and the dilemmas they face. In addition, we wanted to examine how leaders experience and manage the challenges of HSE and QPS in a holistic way.

**Design/setting:**

The study was designed as a case study. Data were collected through semi structured individual interviews with leaders of nursing homes in five Norwegian municipalities.

**Participants:**

13 leaders of nursing homes in urban and rural municipalities participated in this study.

**Results:**

Data analysis resulted in four themes explaining how leaders of nursing homes manage the dual responsibility of HSE and QPS, and the approaches they take and the dilemmas they face:

1. Establishing good systems and building a culture for a work environment that promotes health and patient safety.
2. Establish channels for internal and external collaboration and communication.
3. Establish room for maneuver to exercise leadership.
4. Recognizing and having the mandate to handle possible tensions in the dual responsibility of HSE and QPS.

**Conclusions:**

The study showed that leaders of nursing homes who are responsible for ensuring quality and safety for both patients and staff, experience tensions in handling this dual responsibility. They acknowledged the importance of having time to be present as a leader, to have robust systems to maintain HSE and QPS, and that conflicting aspects of legislation are an everyday challenge.

## 1 Introduction

Healthcare leaders have a complex role and face demands and responsibilities for maintaining the safety of both employees and patients and ensuring high quality care. In particular, healthcare leaders face an important task in managing and handling the dual responsibility of managing Health, Safety and Environment (HSE), and Quality and Patient Safety (QPS), within their healthcare organizations which encompasses both the safety of staff, and the safety and quality of care (1, 2). In Norway, for instance, different legislation regulates these dual responsibilities. Moreover, leaders face a range of practical challenges in the management of QPS including the need to ensure appropriate leaderships skills and system efficiency (3). In the context of nursing homes, there is a pressing need to increase and develop the knowledgebase for the management of QPS (2, 4). The systematic management of HSE and QPS is a leadership responsibility at all levels of the Norwegian healthcare system (5, 6). And, while HSE and QPS are often handled as separate management responsibilities, research shows that it is important to understand HSE and QPS in a holistic and integrated way (1, 7, 8). Managing the duality of safety and wellbeing for both patients and staff is increasingly important for healthcare leaders, and this depends on careful attention to the organizational, cultural, and psychosocial factors that enable safety, but limited attention has been paid to leaders’ understanding and perspectives of this dual responsibilities (1, 7, 9). Effective leadership can build organizational capacity to improve employee and patient outcome, but since leadership is contextual, there can be several ways to lead. Leadership level and organization could affect leadership approach and outcome for HSE and QPS (10, 11). There is some research in hospital settings on mid-level leaders and their role in QPS (12–14), but we know little about how mid-level leaders experience and approach the dilemmas of having the dual responsibility of HSE and QPS. There is limited research on leaders of nursing homes (which, for the purposes of this study, are considered mid-level leaders) experience on HSE and QPS, and this study will contribute to narrow the knowledge gap.

Systems Engineering Initiative for Patient Safety (SEIPS) offers one way of exploring the dual responsibility of QPS and HSE. SEIPS can be used as a theoretical framework that focuses on systems design, and its impact on safety processes and outcomes in healthcare (15). There are five components to a work system in the SEIPS model: person, task, tool/instrument, physical environment, and organizational conditions (15, 16). These components interact with and influence each other in ways that shape safety outcomes. Outcomes in the SEIPS model are divided into patient outcomes and employee and organizational outcomes. Patient outcomes in the SEIPS model are focused on issues related to patient safety or quality of care, while employee or organizational outcomes emphasizes job satisfaction, job stress and burnout, employee safety and health, turnover and profitability (15, 16). The SEIPS model shows how all parts of an organization affect and depend upon each other. By using the SEIPS model for mapping and understanding how nursing home leaders enact their leadership responsibility for HSE and QPS this article contributes to reduce the knowledge gap on how mid-level leaders handle the dual responsibility in their everyday work.

### 1.1 Aim and research question

The aim of this study was to explore how nursing homes leaders manage the dual responsibility of HSE and QPS, and the approaches they take and the dilemmas they face in this work. We wanted to examine how these leaders experience and manage HSE and QPS in a holistic way, and the support available or needed in the process of handling the dual responsibility.

The following research question guided the study:

How do nursing home leaders experience and manage HSE and QPS in a holistic way and what are the key challenges and enablers in this work?

## 2 Methods

### 2.1 Design

**Figure 1:**
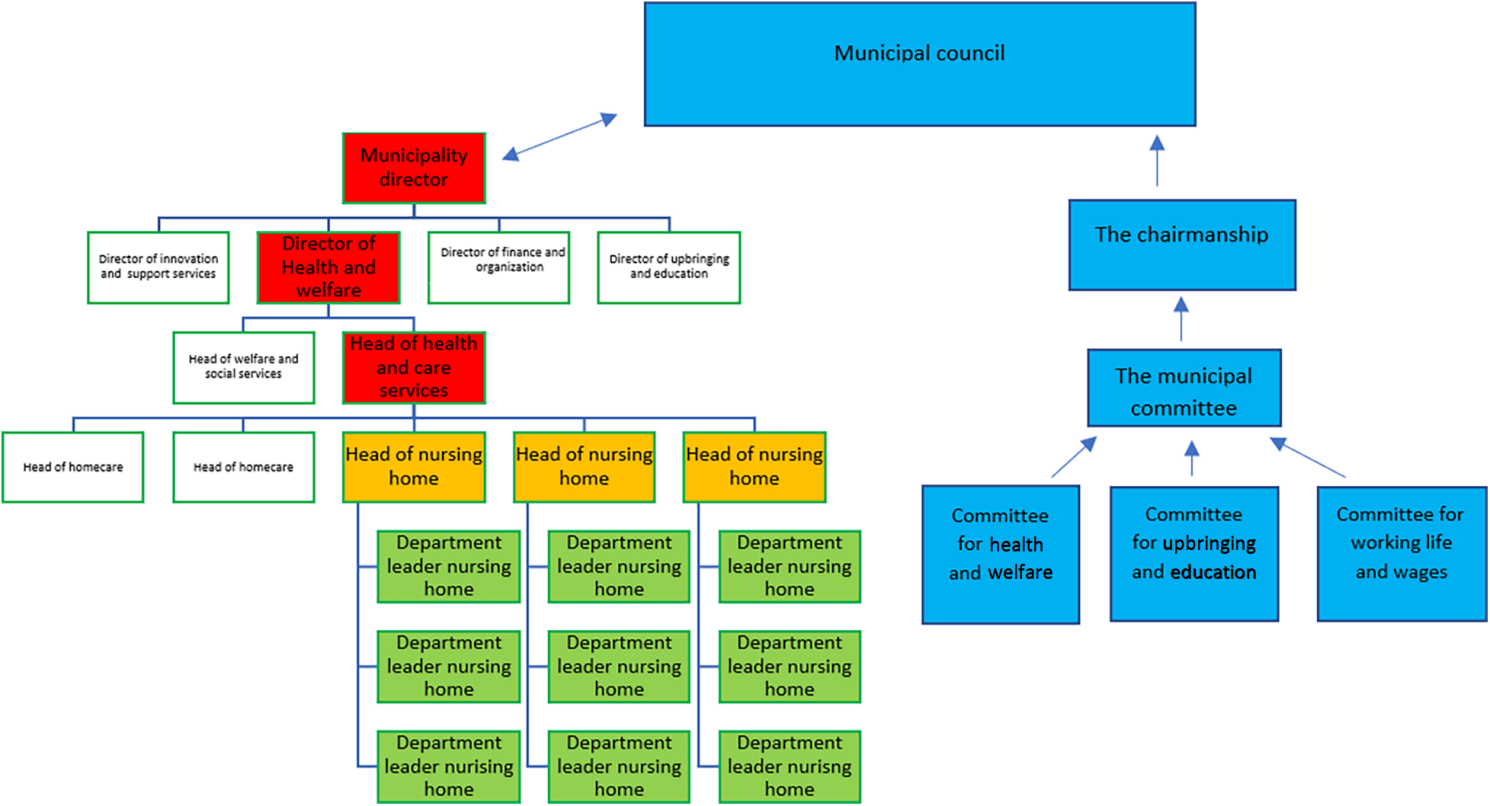
Organization of the municipality according to the chairmanship model. The political steering line is the blue part, while the rest of the organization chart represents the administration. The leadership level relevant in this study are the yellow.

This study is a part of a single embedded case study. A case study is a method that investigates a contemporary phenomenon in depth in a real-world context, and the approach is suitable when wanting to understand a complex social phenomenon (17). In this study the case is defined in terms of the management of two versions of safety: HSE and QPS in Norwegian nursing home context; and how the management of these aspects of safety are organized, controlled, and overseen, particularly considering the possible tensions between them from a leadership perspective. The main overarching research of which this study is one part explores leadership at three levels of the healthcare system, which includes: politicians and top-level leaders in health and care services in the municipalities (municipal director, director of health and welfare, and head of health and care service), head of nursing homes (mid-level leaders), and department leaders in nursing homes (frontline leaders). This sub-study is exploring the experiences of head of nursing homes in five Norwegian municipalities (see Figure 1).

We used the SEIPS model as a framework when formulating the research question and interview guide, and this provides a theoretical reference point throughout the discussion of the findings.

### 2.2 The Norwegian healthcare context; regulation and legislation

Responsibility for public health and care service in Norway is divided between municipalities and regional health care services. Municipalities are responsible for primary healthcare services, which includes home care services, nursing homes, emergency rooms and General practitioners (GP). The chairmanship model is the most common organization of Norwegian municipalities (see Figure 1). In the chairmanship model, political committees have the overall control and management of the municipality, and the municipal council is the highest political authority and makes decisions on behalf of the municipality. Within the legislative framework, the municipalities are free to prioritize and organize work, and develop plans and governing documents. According to the chairmanship model, healthcare leaders must follow the politicians' decisions regarding priorities and budget allocations. Municipalities are regulated by the “Act on Municipalities and County Municipality” (18). The management of QPS is regulated in the “Regulations on management and quality improvement in the health and care services act”, “Health and care services act” and “National action plan for patient safety and quality improvement” (6, 19–21). The management of HSE is regulated in “Regulations on systematic health, environment and safety work in companies” and “The working environment act” (6, 19).

### 2.3 Sample and recruitment

The municipalities were recruited through recommendations from the Norwegian Association of Local and Regional Authorities, of which all Norwegian municipalities and county councils are members. The recommended municipalities were contacted with an invitation to participate in the study, together with an information letter. The municipalities were selected based on size and location (urban/rural). When a municipality agreed to participate in this study, the head of health and care services or an HR-adviser identified and invited potential participating nursing homes with the accompanied information letter on the voluntary participation into the research (see Table 1 for the characteristics of the participating municipalities). Nursing home leaders in the urban municipalities were responsible for a larger number of employees and patients, while leaders of nursing homes rural municipalities were more often responsible for a wider professional field, e.g., nursing homes and home care.

**Table 1:** Characteristics of municipalities and data collection.

Municipality	Urban/rural	Citizens	Number of nursing homes	Interviews
1	urban	42,000	6	5
2	urban	72,000	10	2
3	rural	2,000	1	1
4	rural	2,000	1	1
5	urban	61,000	5	4

### 2.4 Data collection

Data collection consisted of 13 individual semi-structured interviews with leaders of nursing homes (*n *= 13). All participants in this study had extensive leadership experience and formal leadership education. All interviews were conducted physically in the respective nursing home from March and June 2022. Semi-structured interviews were conducted by the first author and were based on an interview guide. Topics in the interview guide were inspired by SEIPS (15), organized in questions dealing with both system-level and individual-level issues, work system, tools, organization, tasks, environment and related to *how leaders experience and manage HSE and QPS in a holistic way, what the key enablers and challenges are, and what kind of structure, system and tools they have in this work.* The interviews lasted approximately one hour. All interviews were audio recorded and transcribed by the first author.

### 2.5 Data analysis

The transcribed data material was uploaded in NVivo and analyzed inductively using thematic analysis. The analysis process followed Braun and Clarkes 6-phase guide (22). See Table 2. Author MRM was responsible for the analysis with input from GSB and SW who red transcripts and discussed theme development throughout the analysis period. The interviews were coded, categorized, and sorted under four main themes on how nursing home leaders experience and manage HSE and QPS in a holistic way.

**Table 2:** Key stages in process of thematic analysis.

1. Familiarizing yourself with the data	Data were transcribed by author MRM and red by authors MRM, GSB and SW.
2. Generating initial codes	Meaning units were extracted, and codes were generated across all data using NVivo by author MRM
3. Searching for main themes	The different codes were sorted into 27 subthemes and potential themes by using thematic map by author MRM
4. Reviewing themes	The extracts were re-read, and subthemes were reviewed by author MRM
5. Defining and naming themes	The essence of each theme was identified, and 4 themes were named by authors MRM, GSB and SW
6. Producing the report	The final analysis was completed, and the report were written by all authors.

## 3 Results

Based on variation in size, organization and number of employees reporting to each leader in each nursing home, the analysis indicated that leaders experienced various challenges and enablers regarding the dual responsibility of handling HSE & QPS. Despite the structural differences in the municipalities and nursing homes, the four identified themes were recurring and common to all the participants. The results are presented theme-wise, with one table for each theme to illustrate initial quotations, sub-themes, and themes. See Tables 3–6.

**Table 3:** Examples of the first theme.

Theme
Establishing good systems and building a culture for a health promoting work environment and patient safety
Sub-themes
System, culture, incidents, full-time culture
Codes
Automatic notification, available system, structure and tools, desire for more structure, system and tools, reporting culture, reasons for poor reporting culture, reasons for good reporting culture, deviation committee, what is deviation, handle notification of deviations, report deviations, report to higher system level, type of deviation, involve employees, rotation, long shifts.
Quote
“Compilo is better than many of the other systems we have had. Here we have both documentation, procedures, and routines, and is where we report deviations. Here we can identify deviations and respond to deviations” (head of nursing home, municipality 1).
“What happened when we became a municipality was that the base of compilo in xx and yy was merged. It wasn't prepared well enough in advance. If you searched for one procedure, you got 8 different procedures. It was chaos and they just merged documents. The result of this was that the employees stopped using compilo. The most important thing for me is that the employees know where to find things, and they 'didn't do that anymore” (head of nursing home, municipality 5).
“There are so many different systems, and computer systems, and they don't communicate…. things could have been simpler and there could have been clearer expectations of what we should do, what is expected of me as head of nursing home” (head of nursing home, municipality 1).
“We have had several challenges because this was a new nursing home with many young employees who had very high competence. We had large positions and only long-term shifts. Then we got another nursing home merged into ours, and of course we had to consider how they had worked and what their everyday life and culture were like. It became two separate departments when they arrived, but still, we are one nursing home and one organization. So, there were quite a few challenges we had to work on”(head of nursing home, municipality 1).
“The night shifts have their own culture, a subculture, where things take place. Getting an overview and grasping it is extremely demanding for us as leaders” (head of nursing home, municipality 5).
“ I report to the head of health and care services. I have a regular meeting every two weeks with her, and deviations is one of the things on the agenda” (head of nursing home, municipality 3).

**Table 4:** Examples of the second theme.

Theme
Establish channels for internal and external collaboration and communication
Sub-themes
Collaboration, organization, overview, expectations, the future, media, the parties in working life.
Codes
The occupational health service, politicians, cooperation with other nursing homes, cooperation with GP, interaction with the specialist health service, supervision, working environment committee, geographical distance between buildings, office location, small municipality, reorganization, organization of work tasks, organization of staff, organization of the nursing home, merged municipality, size of nursing home, overview of deviations, overview of area of responsibility, overview of the consequences of change, expectations from next of kin, feedback from patients and relatives, rethink, future challenges, media, representative of the union, safety representative.
Quote
“I really feel that you do as you want and are quite alone” (head of nursing home, municipality 5).
“When union representatives come to a meeting, they are concerned with their own working environment, they are not concerned with the leaders' working environment. They are concerned with the daily operations, they are concerned with patients, they are concerned with HSE, and they are concerned with the things that matters for them. It is not always that they are engaged in what takes place at a higher level” (head of nursing home, municipality 5).
“The department leaders don't have personnel responsibility, I do. They have more professional tasks and participate in daily operation, so we must reorganize if they are to have time for personnel responsibilities” (head of nursing home, municipality 3).
“I have great faith in the model we are building now, where the nurses become a team that delivers services to the entire nursing home, and then it is the healthcare workers who run the departments to a much greater extent. The healthcare workers gain a higher reputation, have pride in their profession, and possess a lot of expertise that we can now utilize. We need to employ some kind of assistants to make the bed, fill linen trolleys etc., so that the health care personnel are used in the best possible way. We have also started to bring in more social educators in the nursing homes, and they have an environmental mindset that the nurses don't have as much of in their education. When we get all these professional groups together it will be very exciting to work. Nursing home patients are much more complex and professionally exciting now, than they were a few years ago, but we have not been very good at conveying that” (head of nursing home, municipality 2).
“I don't have an overview of everything. I'm a bit of a control freak, so I find it difficult that I don't have a complete overview of all the areas I'm responsible for, but we've created systems that take care of most things and I have the department leaders to help me. Without confidence that they handle their tasks I would not have survived in this job” (head of nursing home, municipality 5).

**Table 5:** Examples of the third theme.

Theme
Establish room for maneuver to exercise leadership.
Sub-themes
Leadership, daily operations, competence and recruitment, sick leave, economy, context, leader status and history.
Codes
To be a leader, unclear expectations of my role, large professional field, independent job, relationship with top-leaders, role and responsibility with HSE and QPS, conflict of loyalty, ask for help, number of personnel responsibility, need for more support, staffing, daily operations, dialogue with employees, sicker patients, basic staffing, lack of time, available doctor, lack of time, resources, blackboard meetings, waiting list, substitutes, need for more training, competence requirements for leaders, have the employees satisfactory competence, sufficient expertise, educational plan, utilize expertise, competence enhancement, recruit nurses, recruitment of managers, reason why it is difficult to recruit, target figure for sickness absence, presence, follow-up of sick leave, arranged work tasks, reduce sickness absence, sickness absence and working environment, sickness absence and quality, return to work, sick pay arrangements, reasons for sickness absence, challenges with sickness absence and QPS, economy, covid, age, experience, education, current position, time with responsibility for HSE and QPS.
Quote
“I don't have enough time to be a good leader. If I had more job opportunities I probably wouldn't be sitting here today, but in small municipalities like this, there is very little to choose from” (head of nursing home, municipality 3).
“It is challenging to have control over everything. We also have the day-to-day operations to take care of, and the way we are organized today makes it difficult because we have so many tasks. It is demanding to practice close leadership, be available and handle urgent matters at the same time as carrying out development work and quality improvement” (head of nursing home, municipality 1).
“We need more staffing. Basic staffing is too low. It works fine if you have completely unproblematic patients and employees who are healthy and present, but that is not everyday life. We have all kinds of situations and challenges, so I would like more staffing” (head of nursing home, municipality 1).
“If you're going to be department leader, you must be a registered nurse, and education in leadership and leadership experience is desirable. It is difficult to hire a leader who does not have this, but the way the market is now, we sometimes must be flexible in what we mean by leadership training and experience” (head of nursing home, municipality 2).
“You are never fully trained, but I feel confident that I can do it. I wish we were more aligned in the municipality, that we talked about what was important and had common guidelines and goals. They have started this process, but it takes time in such a large organization” (head of nursing home, municipality 5).
“I think it's more about time… time to familiarize yourself with all the different systems and tools. it's probably mostly about that, I think” (head of nursing home, municipality 1).
“It is a heavy job, and people talk a lot about it being a difficult profession. Trade unions often talk about it being a difficult profession, and the magazine of the Norwegian Nursing Association writes exclusively negative articles about being a nurse; “running away from the profession”, “can't take it anymore”, “tired” and so on…..and the head of the union says that we must have better wages, we must have better conditions and such….and that…some of my employees says; “I'm so tired of hearing that I'm not well, I feel fine”. We are simply talked down and degraded. You can always find something that is not good, and then you focus more on that than what is good. Learning to turn it around is quite difficult” (head of nursing home, municipality 3).
“If you enjoy your work and you are liked by your colleagues, you will return faster after a period of sickness than if you feel that you are not appreciated by your colleagues, or there are conflicts or dissatisfaction with the leader, and you feel that you are not appreciated. I think there is a big risk that the sick leave will be longer then. I think that we leaders have something to work on here….colleagues too. I think our room for maneuver lies here in relation to being able to influence the working environment” (head of nursing home, municipality 2).
“It's all about economics. There is a requirement to keep budget at the same time as we have statutory tasks to deliver on, and it is a mismatch. I can't keep budgets, I don't think anyone does, unfortunately, and it's a bit demotivating. I don't have the financial resources to be able to have employees who work with quality and professional development, because if I'm going to spend money on that, I must take it from somewhere else and I can't do that” (head of nursing home, municipality 1).

**Table 6:** Examples of the fourth theme.

Theme
Recognize and have the mandate to handle possible tensions in the dual responsibility of HSE and QPS
Sub-themes
HSE and work environment, patient safety, quality, legislation, the dual responsibility, holistic work, the whole picture.
Codes
Conflicts, interpersonal relationships, notifications, standard in nursing homes, work with the work environment, improvement work, follow up internal control, the concept of HSE, employee survey, risk analysis in the event of changes, priorities in the event of conflicting interests, work with patient safety, the patient safety concept, quality work, the concept of quality, measure quality, right quality, standard of quality, coercion, which regulations apply, separate professional fields, holistic work, the concept of the dual responsibility, HSE and QPS are linked, follow up work with HSE and QPS, known issue, what makes working with HSE and QPS challenging, conflicting interests between HSE and QPS, what can make work easier, large area, having the dual responsibility, changes in one field affect the other, HSE and QPS affect each other, connection between the working environment and QPS, relationship with politicians, do the politicians have insight, insight and understanding of managing the municipality, trust from the municipal management, think holistically in the municipality.
Quote
“It's about a lot, but I think it's the working environment and not least sick leave, and sick leave follow-up. It is like the entire content of the nursing home every single day. The most important thing is the working environment, both the psychosocial working environment but also the physical working environment. And then there is sickness absence…as a leader, I am very, very concerned about sickness absence and stability…because it affects everything. How many people we have at work at any given time, well-being, manners, yes, everything…” (head of nursing home, municipality 5).
“Patient safety is that the patient receives services of good quality, and that they are confident they will get what they have resolution on, and that they will have a good life regardless of whether they are in nursing home or receive home care” (head of nursing home, municipality 3).
“Quality is whether the services are performed in relation to requirements and legislation. It is, after all, an indicator of whether there is quality in what you do. But quality can also be experienced… it is a subjective experience… so it can be experienced as good quality for one and bad for another” (head of nursing home, municipality 1).
“With quality, I think we have an extremely large amount of legislation. Putting it into use and into practice can be very difficult for many and making it simple enough so that the employees know what to do and know that quality is regulated by law is important. We have professional ethical guidelines, we have an extremely large number of other things that it is important that we as employers comply with to ensure quality. I could talk about this for a long time. You have the Norwegian National Insurance Act which says something about not working more than 100%, and if you do so, you must tell your employer because we must make sure that the employees at work have the surplus to look after the patient. For me, these things are quality” (head of nursing home, municipality 5).
“It is very difficult to measure. We can do it…not well enough, but we have some system for internal control where we can go in and take random samples of documentation, whether you document and report on the same things and that you follow up on what is documented. we have a deviation system that gives an indication of where the failure is, why it fails, what can we do, are there routines we need to change, are there people who can’t, don’t know, don't want to? So that we can follow up. It gives us an indication of quality, and if there are many deviations in an area, it gives us an indication that things are not working” (head of nursing home, municipality 5).
“It is difficult in the health sector because we have an enormous amount of legislation that we must deal with. It is very easy to get caught up in it and to focus a lot on certain things, which makes it more difficult to see the whole” (head of nursing home, municipality 5).
“Every day we have a balancing act between the use of coercion on patients and safety for staff. We spend a lot of time building competence among the staff so that they are safe” (head of nursing home, municipality 1).
“Within dementia care there is always a balance between the use of coercion and safety for staff. It is about building competence among staff. We see very clearly how important competence is, that a working environment is good and safe, that they know what tasks they have and that they work well together and have trained on different situations” (head of nursing home, municipality 1).
“When I start thinking about it, it's terrifying. The most frightening thing is perhaps the people living at home, it is the part you have the least overview of, in the homes and various arenas there. It is perhaps the responsibility that weighs the most because we can have a patient who only has a decision on medicine management, for example, that they can have a medicine dispenser, and when the alarm goes off that they have not taken their medicine, the staff moves out. Not long ago we had an incident where the person in question had not taken his medicine and then a healthcare worker went out. She did not find the user at home, but there was a room she did not enter, and there the user was lying and had suffered a stroke. And it was….the relatives had expected her to have checked the whole house, and….it is frightening to know that it was expected….it is a huge responsibility and therefore it is important that we have good routines. When this incident happened, we sat down in home care and went through the situation and looked at what we could do to prevent this from happening again. Now it is routine that you should at least go into the bedroom. Great demands are placed on us. We must ensure good routines and procedures, and that everyone knows them. although one can never secure against everything. But it is perhaps this that weighs the most. But I still sleep well, otherwise I couldn't have this job. But you must trust the people who work here” (head of nursing home, municipality 3).
“Of course, the psychosocial working environment is one of the things that affects the quality of our services. A good working environment alone does not ensure good patient safety. So much more needs to be done, but when we see what a good psychosocial work environment is…cooperation, respect for each other, exchange of…..a sharing culture, what have I done well, what have you done well….of course it affects each other” (head of nursing home, municipality 1).
“I feel it is a bit ad hoc and coincidence that governs many of the local politicians and how they exercise their role. they handle individual cases and forget that they are ultimately the employer and that they are the ones who determine the financial framework” (head of nursing home, municipality 1).

### 3.1 Establishing good systems and building a culture for a health promoting work environment and patient safety

For all leaders, having good and sufficient work systems was deemed necessary to have an overview and degree of control over HSE & QPS in the nursing home. They also indicated that having a culture for using the available systems and reporting adverse events was essential for a safe work environment for both patients and staff.

#### 3.1.1 System

Leaders who were responsible for more than one nursing home had a greater need for management systems and structures than those who were responsible for one nursing home only. The size of the nursing home was also important, as participants who were responsible for large nursing homes with many departments needed more sophisticated management systems to maintain overview and control of safety. All nursing homes in the sample used Compilo as their quality system. Compilo is an electronic system where documents can be stored. It is moreover used to save updated regulations, guidelines, and procedures, and for reporting deviations, such as adverse events. There was great variation in the extent to which the participants used the various components of Compilo, but the majority were satisfied with the functionality the system provided. One of the participating municipalities was newly established, merged from two smaller neighboring municipalities. The two original municipalities were described as having had both different systems and a different culture around safety. The leaders experienced that the systems were not easily adapted to each other when the municipalities were merged, with the result that the employees stopped using it. The leaders subsequently spent a lot of time establishing a management system that works for everyone and encouraging a culture of engaging with the new system. The participants experienced that they had many and good systems, but the systems did not communicate with each other, and leadership expectations were unclear.

#### 3.1.2 Culture

Cultural issues were often invoked to explain challenges and opportunities within both domains of HSE and QPS, and the nursing home leaders argued that culture influenced several areas. To build a culture for a health promoting working environment and patient safety, the nursing homes must build a common culture regarding how work is organized, the reporting of adverse events, the approach to working hours, as well as building a shared sense of coherence and community, the leaders said. Reorganizations and merger processes in recent years have led to several nursing homes being merged, rebuilt, or newly established. Leaders of merged or newly established nursing homes talked about the importance of working towards a common culture among their organizations and employees.

#### 3.1.3 Incidents

Generally, the leaders believed they had been able to establish a good culture for reporting deviations and incidents. Incident reporting was a focus area, and leaders described that improvements in reporting culture required ongoing leadership emphasis on the importance of reporting. Some nursing homes and departments had a less effective reporting culture than others, and the leaders explained this was due to a historic lack of attention to developing reporting systems, staff being fearful of reporting incidents involving their colleagues, and a lack of training in how to use the reporting system. Leaders described how they encouraged the employees to report and subsequently used the incidents to support improvement work, however it was often deemed challenging to define what exactly constituted a reportable deviation in the context of nursing homes, and leaders suspected those uncertainties could lead to underreporting. The incidents reported mainly concerned events immediately related to patient care, such as medication errors and falls. Some were related to threats and violence from patients and next of kin, while there were almost no reports on more contextual or organizational issues such as the work environment. The incident reports were handled by the department leaders. To a varying degree, leaders in a higher hierarchical position were informed. Information about deviations was not requested by the top leaders in the large municipalities, while leaders across leadership levels in the small municipalities had regular meetings where deviations were on the agenda.

### 3.2 Establish channels for internal and external collaboration and communication

To meet the future needs and expectations from society in HSE and QPS, leaders of nursing homes saw the need for clear communication and cooperation with others. The degree of interaction varied in the different municipalities, but all had routinely collaborated with other stakeholders such as specialized healthcare services, the occupational health service, and GPs. Some leaders experienced effective collaboration with other leaders in the municipality, while others reported working more independently without the aid of common guidelines and close professional interaction. Collaboration with the governmental authorities through formal process of regulatory supervision was perceived by most leaders as constructive, and a way for the leaders of nursing homes to identify and focus attention on improvement activities. Politicians and media were seen as playing an important part in highlighting important safety issues, as well as communicating broader expectations, challenges, and opportunities according to the leaders.

#### 3.2.1 Organization

The participating municipalities differed in size and organizational structure. In the larger municipalities there were several department leaders with personnel responsibility, while in the small municipalities, the department leaders did not always have personnel responsibility, and were more preoccupied with daily operations and rotation scheme. Employees within the nursing homes were organized in different ways. In some nursing homes they belonged to a specific department and were not flexible about changes, while in others, staff were used to work across departments to balance workload and resource. With greater flexibility in staffing, leaders found it easier to cover vacant shifts with equivalent staff and make less use of temporary staff. This was beneficial financially as it reduced costly reliance on temporary workers, and allowed leaders to ensure that teams with the appropriate skills and competence could be flexibly reorganized as necessary. The leaders who organized staff in this way initially experienced reluctance from some team members, but said that with guidance, support and a gradual cultural change, employees acknowledged the positive effect for both HSE and QPS. Leaders also indicated that they expected that the need to organize work in new ways, would only increase to meet future needs, such as the increasing number of elderly people in need of complex and advanced care.

#### 3.2.2 Overview

The leaders of nursing homes reported that it could be challenging to maintain an overview of everything they were responsible for in relation to HSE and QPS, and described the need for effective systems, collaboration, and communication. Collaboration with representatives from the unions and safety representatives was seen as essential for leaders to have a clear view of health and safety issues in the work environment, to implement improvements and to create rotation schemes that consider both employees’ wishes, the need for competence and continuity in care, and laws and regulations on working hours. Although leaders valued this engagement, they also reported that union representatives did not always see the whole picture in the same way or were concerned with the leaders’ working conditions.

### 3.3 Establish room for maneuver to exercise leadership

All participants in this study had extensive leadership experience at different levels in the health care system. They agreed that it was challenging to find time and room to fully perform their leadership duties, as daily operations, financial limitations, and sick leave took considerable time and attention. They acknowledged the need to establish room for maneuver to ensure time and resources for practicing the leadership responsibilities.

#### 3.3.1 Leadership

Being a leader was described as hard work. Leaders found it challenging to always be available to address challenges, and commonly reported that the distinction between work and leisure had been blurred, and that, particularly in the small municipalities, they could feel lonely in relations to their leadership and organizational responsibilities. Daily operations took time away from being physically present, and several of the participants found it challenging as they had responsibility for several nursing homes, and some employees worked nightshifts or weekends only. Participants highlighted the importance of practicing leadership by “being near” the employees. As leaders of nursing homes, they felt an enormous responsibility for both their patients’ and staff safety and wellbeing.

#### 3.3.2 Daily operations

Daily operations took up much of the leader's time and availability, which resulted in more development-oriented work and long-term planning not being prioritized. Nursing home patients have become sicker and more demanding compared to a few years ago and leaders felt that the lack of resources available to them had not kept pace to handle this development. The staffing situation was described as similar, which could affect the management of both HSE and QPS in terms of not having enough qualified personnel. The leaders suggested implementing minimum staffing levels to maintain safe care.

#### 3.3.3 Competence and recruitment

Staff competence was viewed as critical for the delivery of safe services and high quality care. Competence was also described as the foundation for meeting future needs and increasing patient demands and keeping health care personnel engaged and self-confident. Leaders reported that recruitment of nurses and department leaders had become more difficult and having substitutes in vacant positions affected daily operations and the overall competence. Heads of nursing homes in this study considered their department leaders to be appropriately qualified, but not all of them had formal leadership training or leadership experience. The leaders reported that they felt they had the competence needed for their own leadership position, but they also acknowledge that there was room for improvement in how the competence was used and maintained. To be a leader in a nursing home, there were no requirements other than a bachelor's degree in a health-related topic. In most municipalities it was required to be a registered nurse, while others also had physiotherapists and social workers as leaders. There were good opportunities for skills development internally in the municipalities through courses and further education regarding HSE and QPS. Despite these opportunities, many participants explained that the lack of time to get used to new systems and routines made everyday life demanding.

#### 3.3.4 Sick leave

Sickness absence affected everyday life in nursing homes to a great extent and required a lot of attention and management by the leaders. Leaders argued that employee age, culture, and sick pay arrangements were factors they believed influenced the high rate of absence. Moreover, they believed that the reputation of working in nursing homes contributed to sick leave, as they experienced work being talked down and employees being told that it was a heavy and demanding profession. Since lack of visible and existing leadership can lead to increased sick leave, this became a negative spiral. Loosing continuity and valuable competence was the biggest consequence of the sick leave. Leaders often had to use unqualified substitutes during times with high sick leave, and several of the leaders tried to reorganize their nursing homes and how they used their staff so that they were not so vulnerable to absence. They all agreed that sick leave affected the working environment, and thus QPS, but also that a good work environment could contribute to employees returning to work earlier and maintaining effective work engagement. The leaders created some flexibility by changing work roles and adapting work demands so that employees could return faster after sick leave. But it was difficult when employee needs or necessary accommodations were long-term, or if there were several employees in need of easier work tasks or changed working hours. The leaders agreed that working with the rotation schemes, work environment, and increasing basic staffing levels would reduce sickness absence.

#### 3.3.5 Economy

Economic considerations influenced several of the areas the leaders were responsible for and was decisive for whether they could hire additional personnel if needed. According to leaders, budgetary constraints affected most of their decisions, and they felt there was a gap between the financial resources they had available, and the tasks and quality of care they had to deliver.

### 3.4 Recognizing and having the mandate to handle possible tensions in the dual responsibility of HSE and QPS

The concept of dual responsibility for HSE and QPS was not a term that leaders were accustomed to. Several participants experienced that it could be difficult to separate the concepts of HSE and QPS. They argued it was holistic work that could not be divided. They acknowledged having an enormous responsibility, and that there were possible tensions within specific legislation and requirements. This affected their management of both HSE and QPS.

#### 3.4.1 HSE and work environment

HSE was seen as a wide area that was difficult to define, but in addition to the physical work environment, the leaders highlighted the importance of the psychosocial work environment. They all agreed that internal control should be used as a tool in associated improvement activities, but not all nursing homes had a system to support this. Employee surveys were also used as a foundation to understand the working environment, and leaders had all put in place for this. Covid-19 contributed to a delay in following up employee surveys due to restrictions and meetings across cohorts.

#### 3.4.2 Patient safety

Patient safety was seen as important and described as safe treatment and care, and the avoidance of injury and inconvenience for patients. Participants furthermore argued that patient safety also was related to more holistic care and dignity. Having routines and procedures was crucial for patient safety, leaders believed. The greatest enablers of patient safety in nursing homes were ensuring that staff follow routines, organizing everyday work effectively and recruiting qualified and appropriate personnel. One of the participating municipalities had participated in a patient safety program a few years ago, and as a result leaders in that location remained particularly focused on patient safety.

#### 3.4.3 Quality

The concept of quality was difficult to define for the leaders. They all agreed that delivering services of high quality was important, but they had no clear definition of what high quality was. Quality was described as being about whether the services are performed in accordance with laws and requirements, but they also believed that quality was a subjective experience and talked about the importance of defining the “right” quality level. A key challenge related to the ability to provide the same level of quality of services throughout the nursing home, and that this could depend on which personnel were at work. Quality and safety influenced each other, participants said. High service quality was an overall objective for the leaders, however, they found it challenging to put this into practice when they did not have a clear definition of high quality and what is “good enough”. Having routines and procedures was viewed as important to maintain quality, but measuring quality was considered difficult across all municipalities, with most making use of user surveys, incident reports and risk analyses to understand quality of care.

#### 3.4.4 Legislation

Legislation and requirements regulate the work related to HSE and QPS to a large extent. Leaders reported that it was difficult to find room to maneuver and make local decisions in relation to the various laws and regulations, especially when these were contradictory. They described an everyday balance between different legislation, ensuring both patient and staff safety and trying to get an overview. Especially the use of coercion on patients and maintaining safety for the healthcare personnel was highlighted as conflicting by the leaders.

#### 3.4.5 The dual responsibility

HSE and QPS were according to the leaders a large field that could be challenging to maneuver in. They experienced this as concepts that influenced each other, and should be seen as one, but often had conflicting interests. To handle HSE and QPS and possible tensions between them participants needed to create good work systems and have confidence in the department leaders and staff that they are competent, uses the systems and report incidents when they occur. They all agreed that HSE and QPS was connected, and especially work engagement and a good working environment were seen as factors that influenced QPS. When changes were made, consequences in other areas were always considered. In events of conflicting interests e.g., use of coercion on patients and safety for staff, the participants had different points of view on what to prioritize. They had no instructions from the municipal management on how to prioritize HSE and QPS concerns in situations where these conflict, but they experienced having support and trust from the municipal management. The relationship with the politicians as decision makers and employer was more complicated as the informants experienced that they were not always supportive and lacked a comprehensive understanding.

## 4 Discussion

**Figure 2:**
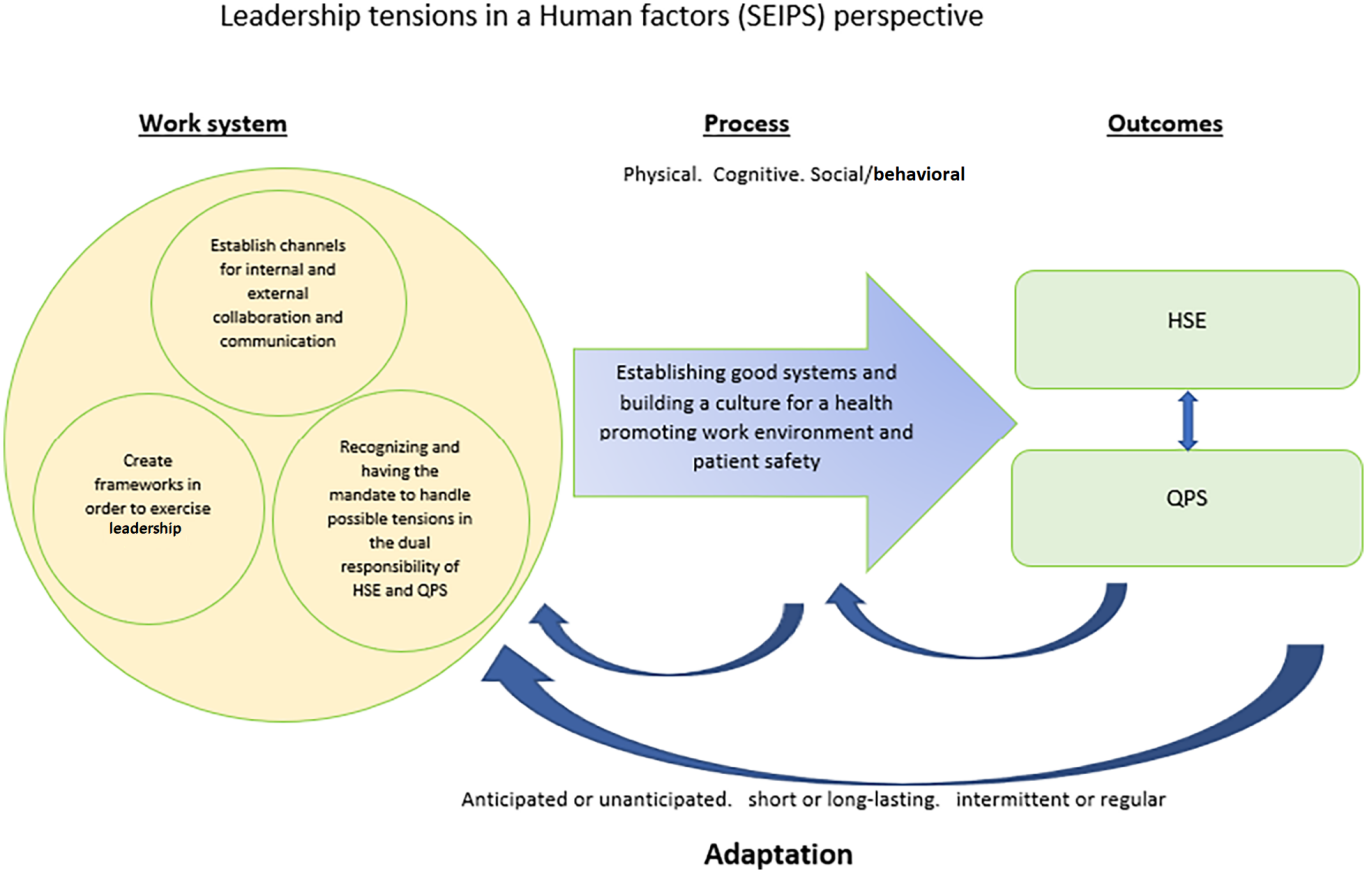
The four themes of how nursing home leaders manage the dual responsibility of HSE and QPS in a human factors’ perspective.

This study explored how nursing home leaders manage the dual responsibility of HSE and QPS, and four identified themes shed a light on how they experience this responsibility, their approaches, and the dilemmas they faced. Our findings show the complexity involved for leaders when enacting the dual responsibility of HSE and QPS, and culture, organization and leadership affect experience and outcome for patients and staff. We have discussed the results of this study considering the SEIPS model and have developed this further in Figure 2 (see Figure 2).

In a SEIPS and Human factors perspective these challenges can be systematically analyzed with a systems orientation to better understand the work system, processes, and outcomes by enacting their leadership roles in a nursing home context. This is discussed in the following along with future implications (23–25).

### 4.1 Leadership and organization

**Figure 3:**
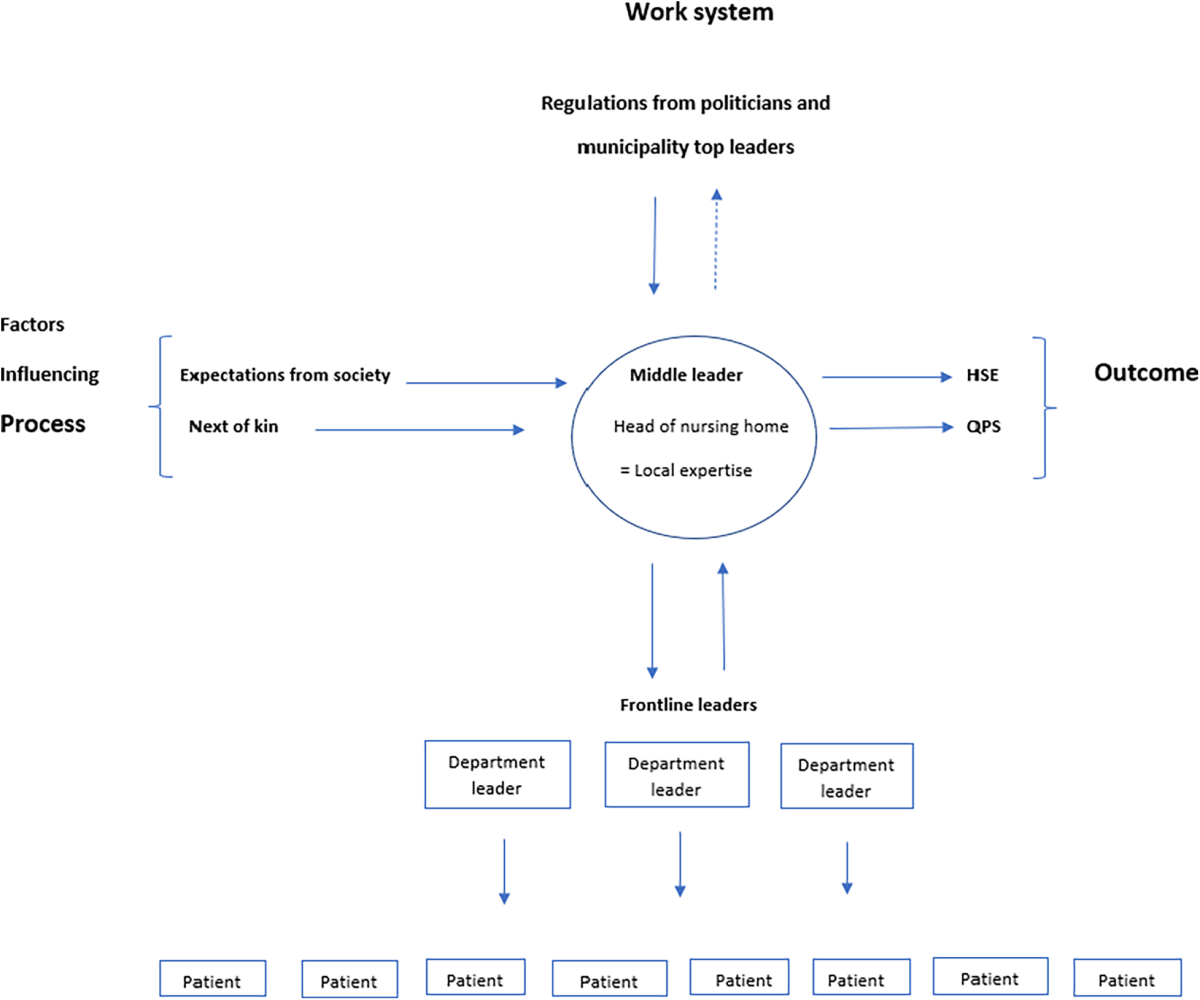
Mid-level leaders position in the municipalities, with responsibilities, considerations and influencing factors in a human factors perspective.

Mid-level leaders are defined as employees that are supervised by an organization's top-level leaders and who supervise frontline leaders and oversee a defined part of the organization (10, 26). Head of nursing homes holds a dual position both as mid-level leaders (within the overall care system) and as top-level leaders (within their specific organizations). As depicted in Figure 1 they enact the role as mid-level leaders because they are situated in the midlevel of stakeholders in the municipalities who are responsible for organizing the primary care services for all inhabitants (see Figure 1). At the same time, they are the top-level leaders within their specific care organization (nursing home) having the highest decision-making authority and responsibility, and employees at the nursing home could see them as their top-level leader. Healthcare mid-level leaders have an important role in translating top-level policies, strategies, and means into sustainable, high-quality healthcare (27, 28). For participants in this study, it was experienced as a challenge, having the highest management responsibility at the nursing home, being responsible for HSE and QPS, while being subject to instructions from the municipal management and politicians and at the same time handling the expectations from society and next of kin (see Figure 3). Mid-level leaders in healthcare are often professionals who have taken on a leadership role with limited leadership qualifications, and support (10, 27, 28). In our study, all participants had a health-related bachelor's degree, leadership education and leadership experience. This gave them a unique position as they know their organizational area well and can translate the strategy of top-level leaders (in the healthcare system) into everyday life (26, 29). Top-level leaders support have a significant effect on mid-level leaders commitment and effectiveness (29), and studies show that mid-level leaders are important for the organization as they can fill structural holes, motivate and engage employees in changes and improvement (10, 26, 30, 31). Our results indicate that leaders of nursing homes felt alone and had limited support from the municipalities’ top-level leaders. This could influence their engagement and ability to inspire employees in day-to-day operations and future research and practice should explore enablers of stronger support structures and processes within organizations, to avoid potential burnout and turnover of employees and its negative effects on QPS and HSE.

Leadership is associated with both employee and patient outcome (32–34), and different leadership approaches may enable different outcomes. Transformational leadership has significant impact on patient safety culture and work engagement (33), and Leader-Member exchange (LMX) theory suggests that leaders can develop high-quality relationships with employees and thus affect safety culture (35, 36). A fundamental managerial function is to ensure that the organization is adapted to its environment (37). Reform processes in Norway (regionalization and mergers) have resulted in frequent reorganizations, and together with future challenges and expectations this requires constant organizational adaptations and attention from the nursing home leaders. According to Carayon and Perry it is important to listen to and understand local expertise, since all work system have different barriers and facilitators (23). In this study, the nursing home leaders had local expertise regarding local opportunities and challenges, while politicians and municipal top-level management made decisions that applied to all nursing homes in the municipality regardless of local needs. In line with SEIPS this shows how both organizational factors within the nursing home, and factors in the external environment such as legislation, the economic situation and policy factors, can affect the work system that leaders are responsible for (i.e., the nursing home) and the people carrying out the tasks (i.e., the employees and leaders of nursing homes) (38–40). Handling the dual responsibility of HSE and QPS while complying with decisions from top-level leaders, cause continuous trade-offs for the leaders in this study. According to the Efficiency-Thoroughness Trade-Off principle (ETTO), there is a constant balancing act between demands and resources that affects efficiency and thoroughness, and an imbalance between these can lead to errors and adverse events (41). Achieving the right ETTO-balance is an important leadership responsibility within QPS and HSE (42, 43). In practice, this study indicates that it is the nursing home leaders who have the local expertise to make appropriate trade-offs to balance any tensions and manage HSE and QPS. However, our results indicate that there is a need for future studies investigating the role of local expertise when mid-level leaders maneuver in a landscape characterized by conflicting demands with the current local situation and policy and guidelines from the municipal management and politicians.

Changes and reorganizations may have negative effect on the working environment (44), and in our study we found that work environment, QPS and sick leave were closely connected. According to legislation, sick leave follow up is a leadership responsibility (6), and our results showed that leaders spent a lot of time on this task. Nursing home leaders experienced that, by investing a lot of time in employees on sick leave, they traded off other leadership tasks. Having time to fulfill all leadership duties was a constant challenge for nursing home leaders, and contributed to a “firefighting” approach, which other studies have shown is not in accordance with how healthcare professionals and leaders conceptualize providing high quality care (2).

### 4.2 Culture and QPS

Leaders in healthcare have a legal and professional obligation to improve and provide high quality care (12). Quality of care is a complex and multi-layered concept that is described in various ways including dimensions of clinical effectiveness, patient safety, patient experience as the most common elements in addition to equity, time, and coordination (45). The participants in this study experienced quality as difficult to define, and agreed that quality seemed to be situated, practical and linked to the actual work and care being done. They found quality to be subjective, and thus difficult to measure. Ensuring feedback loops from the organizational context and work system is important for learning, improvement and adaptation (16).

Organizational culture may affect how efficiency-thoroughness is negotiated and balanced, and thus affect QPS (43, 46). Leaders in this study were concerned that certain cultural characteristics might adversely influence multiple areas in the organization and affect work associated with HSE and QPS. Leadership is a key factor in creating, encouraging, and developing safety culture, and it is important that there is not too much pressure on mid-level leaders and that they have the right resources available (47, 48). In our study, nursing home leaders felt an enormous pressure from both policymakers, top-level leaders, frontline leaders, and society (see Figure 3), and they all described challenges related to a lack of resources (e.g., economy, qualified personnel, and support). As a way of utilizing resources, reducing sick leave and improve continuity and quality of care, the nursing homes had introduced full-time culture with long shifts. Studies show that long shifts could lead to unintended consequences such as burnout, reduced efficiency and effectiveness, and thus hinder QPS (49). SEIPS 2.0 states that changes in work system (e.g., long shifts), could have a delayed effect on outcomes (e.g., fatigue and turnover) and lead to a higher level of risk and more deviance (38).

In the event of conflicting interests between HSE and QPS, the leaders had different preferences on what should be prioritized, and there were no instructions from the top-level leaders. In line with other research (1, 12), leaders indicated that HSE and QPS were connected, and work engagement and a good working environment were considered as factors positively influencing QPS. Healthcare leaders possess an important role in QPS, and there is clear evidence that leaders have a great impact on workplace safety and organizational climate. This demonstrates how HSE and QPS should be understood in a holistic perspective (1, 7, 8). From a human factors and SEIPS perspective different priorities (process), will give different outcome for patients and staff and more research is needed to get in depth knowledge in this field.

### 4.3 Strengths and limitations

This study is the first to explore nursing home leaders’ experiences of the dual responsibility of HSE and QPS and associated key enablers and challenges. The strength of this study is that it contributes new insight regarding the challenges faced by nursing home leaders when handling this duality. The experience and challenges explored here are not exhaustive, but they provide insight that may be transferrable to other similar contexts (17). The study has some limitations. The participating leaders were recruited by the municipal top-level leaders, with some risk of selection bias or unintended pressure to participate in the study. Participants were informed both verbally and in writing that they could withdraw from the study at any time. When using a semi-structured interview guide, it is possible that participants may be prompted to answer in a certain way (50). To minimize that risk, we informed participants that we were interested in their experiences, and that no answer was right or wrong. This study consisted of 13 participants, and having a higher sample of leaders and municipalities could have provided other elements to the results.

## 5 Conclusions and implications

This study shows that both contextual factors and internal factors influence how nursing home leaders experience the dual responsibility of HSE and QPS. Nursing home leaders reported that time to exercise visible and present leadership, systems to maintain HSE and QPS work, and that conflicting legislation influenced their experience and ability to manage the dual responsibility of HSE and QPS. This study confirms that a change in one of the system components, e.g., organization of the employees (the work system), may affect how the work is carried out due to economic status, available and qualified personnel, etc. (outcome). We have theorized leadership tensions from a human factors perspective to develop a better understanding of nursing home leaders maneuver to handle the dual responsibility of HSE and QPS particularly in relation to the organization and context of Norwegian municipalities. Research on the dual responsibility of HSE and QPS is limited in a nursing home context, and more studies should be conducted to explore the middle leaders experience.

## Data Availability

The raw data supporting the conclusions of this article will be made available by the authors, without undue reservation.
